# *Staphylea bumalda* Alleviates Dextran Sulfate Sodium-Induced Ulcerative Colitis in Mice by Regulating Inflammatory Cytokines, Oxidative Stress, and Maintaining Gut Homeostasis

**DOI:** 10.3390/molecules29215030

**Published:** 2024-10-24

**Authors:** Lu Wang, Sha Long, Qi Zeng, Wanrong Dong, Yaoyao Li, Jiangtao Su, Yuxin Chen, Gao Zhou

**Affiliations:** 1 Hubei Key Laboratory of Industrial Microbiology, Key Laboratory of Fermentation Engineering (Ministry of Education), Cooperative Innovation Center of Industrial Fermentation (Ministry of Education & Hubei Province), Hubei University of Technology, Wuhan 430068, China; wanglu611@foxmail.com (L.W.); longshahbut@foxmail.com (S.L.); zqi99@foxmail.com (Q.Z.); dongwwwrr@163.com (W.D.); yyl030425@163.com (Y.L.); jiangtsu@hbut.edu.cn (J.S.); yuxinc@hbut.edu.cn (Y.C.); 2 National “111” Center for Cellular Regulation and Molecular Pharmaceutics, School of Life and Health Sciences, Hubei University of Technology, Wuhan 430068, China; 3 Post-Doctoral Research Center of Mayinglong Pharmaceutical Group Co., Ltd., Wuhan 430064, China

**Keywords:** *Staphylea bumalda*, ulcerative colitis, inflammatory factor, oxidative stress, gut flora homeostasis

## Abstract

*Staphylea bumalda* is a rare medicine and edible shrub native to the temperate regions of Asia, possessing significant medicinal potential. In this study, the components of *S. bumalda* tender leaves and buds extract (SBE) were analyzed and identified by HPLC and LC/MS method, and the safety of SBE was evaluated through mouse acute toxicity models. The protective effects of SBE on dextran sulfate sodium (DSS)-induced ulcerative colitis (UC) in mice were investigated in terms of inflammatory factor levels, oxidative stress, and gut microorganisms. Results showed that hyperoside, kaempferol-3-O-rutinoside, isorhoifolin, and rutin were the main components of the extract, and SBE demonstrated good safety in experimental mice. SBE could alleviate weight losing, disease activity index (DAI) raising, and colon shortening in mice. Pathological section results showed that the inflammatory cell infiltration decreased significantly, and the number of goblet cells increased significantly in the SBE group. After SBE treatment, interleukin-6 (IL-6), interleukin-1β (IL-1β), and tumor necrosis factor-α (TNF-α) levels in serum were significantly decreased, and the levels of myeloperoxidase (MPO) and nitric oxide (NO) in colon tissues were significantly decreased. SBE inhibited gut inflammation by increasing *Lactobacillus*. In summary, SBE played a therapeutic role in UC mice by relieving colon injury, reducing inflammatory factor levels, and maintaining gut flora homeostasis. SBE is expected to become an auxiliary means to participate in the prevention and treatment of UC.

## 1. Introduction

*Staphylea bumalda* DC. is a perennial deciduous shrub belonging to the genus *Staphylea*, has high ornamental value because of its pearl-like color, and is also known as the pearl flower [1]. *S. bumalda* is mainly distributed in temperate areas of Asia such as China, Japan, and Korea [2]; specifically, it is widely distributed in Henan, Zhejiang, and Hubei provinces in China [3]. *S. bumalda* is a rare medicinal and edible shrub with a long history in the Dabie Mountains, China [4]. According to the “Chinese Materia Medica”, *S. bumalda* is used as a medicine with the fruit and root to moisten the lungs and relieve cough, which has certain medicinal value. Studies showed that its tender leaves and flower buds were rich in flavonoids, calcium, iron, selenium, and other nutritional elements [5]. *S. bumalda* is a kind of woody plant that can be used as healthy vegetable and new medicinal resource, and it has high commodity value and market prospects. The flavonoids contained in *S. bumalda* [5] have good potential for anti-inflammatory [6,7] and antioxidant [8] activities. Plant flavonoids, as scavengers of reactive oxygen species and reactive nitrogen species, have certain antioxidant activity and can inhibit the release of inflammatory mediators. In addition, a complex relationship exists between flavonoids and gut microorganisms, which can maintain gut homeostasis in terms of type and quantity [9,10].

UC is a recurrent inflammatory bowel disease with typical clinical symptoms such as hematochezia, diarrhea, and abdominal pain [11]. It has become a global disease that threatens human health. According to recent surveys, the prevalence of UC has been steadily increasing in developed and newly industrialized countries [12], and its causes are complex [13]. UC is generally believed to be caused by a combination of genetic susceptibility, environmental factors, gut barrier defects, and abnormal immune responses [14]. Considering the chronic nature of the disease, long-term and often expensive treatment is required, such as traditional intervention drugs, including 5-aminosalicylic acid, and steroid hormones [15]. They have good short-term effects, but their long-term efficacy is poor, and the recurrence rate is high after withdrawal [16]. Although treatment options are expanding, 10–20% of patients still need to be treated with resection, which is painful and expensive to treat. Therefore, finding natural anti-UC active substances that are safe with high efficiency and low side effects is particularly important.

In recent years, the treatment of medicinal and food homologous natural products in the field of anti-inflammatories has attracted wide attention domestically and overseas. For example, plant polyphenols have been proven to achieve gut protection by reducing pro-inflammatory cytokines and promoting the growth of gut probiotics [17,18]. In addition, studies have shown that natural products of flavonoids have obvious therapeutic effects on UC through anti-inflammation, which promotes mucosal healing, maintains gut immune homeostasis, and regulates gut flora [19,20]. We investigated the potential application of *S. bumalda* in the treatment of UC using a gut disease model. This study aimed to provide new ideas for the development of *S. bumalda* and offer dietary guidance for UC patients with it as a new type of natural medicinal plant.

## 2. Results

### 2.1. Qualitative and Quantitative Analysis Results of the Main Components of SBE

**Figure 1:**
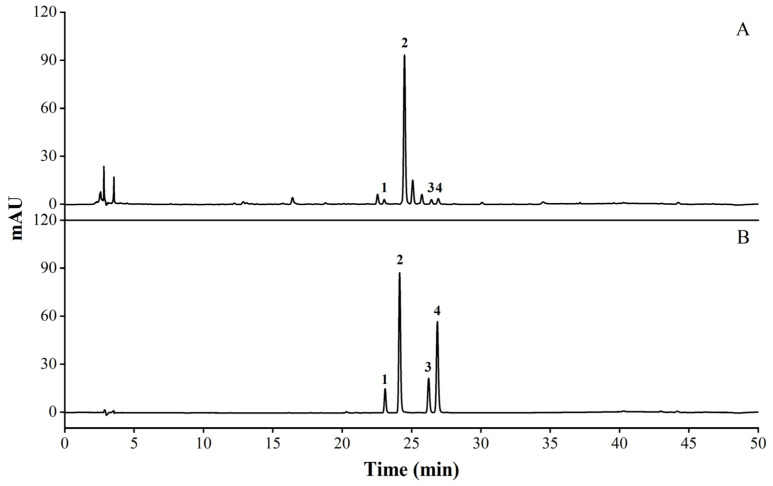
Results of comparing the specific standard with the composition in the sample by HPLC. (**A**) SBE; (**B**) standards (1. rutin; 2. hyperoside; 3. kaempferol-3-O-rutinoside; 4. isorhoifolin).

LC-MS analysis was performed on SBE, and 19 compounds were inferred, including various flavonoids. The analysis results are shown in Table 1. According to the results of LC-MS, standard products were compared by HPLC under specific conditions and wavelengths. The flavonoids contained in SBE were mainly hyperoside, kaempferol-3-O-rutinoside, isorhoifolin, and rutin, among which hyperoside had a maximum absorption peak at the wavelength determined by 254 nm. The contents of hyperoside, kaempferol-3-O-rutinoside, isorhoifolin, and rutin in SBE were (8.52 ± 0.82)%, (1.54 ± 0.14)%, (0.91 ± 0.06)%, and (0.34 ± 0.03)%. The methodological research is shown in Table S1. The results of components compared by HPLC are shown in Figure 1.

### 2.2. Effects of SBE on Acute Toxicity in ICR Mice

**Figure 2:**
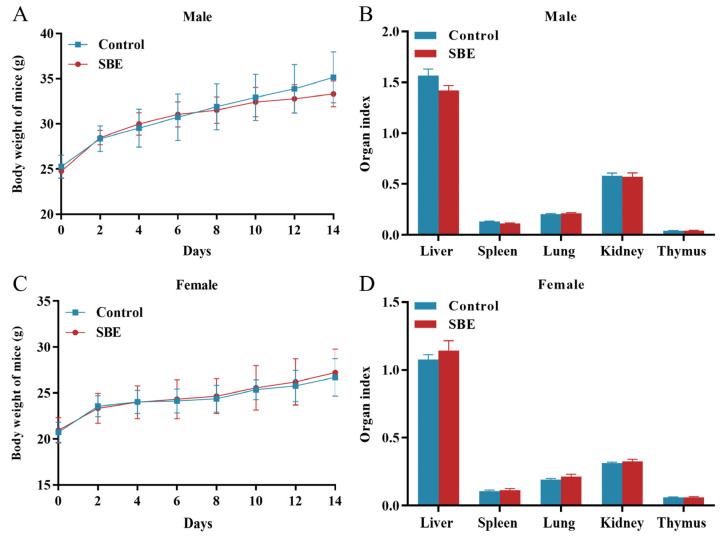
Mouse acute toxicity experiments of SBE (20 g/kg). (**A**) Changes in body weight of male mice; (**B**) organ index of male mice; (**C**) changes in body weight of female mice; (**D**) organ index of female mice (*n* = 10).

The mice were observed continuously for 4 h after oral gavage administration; it was found that the mice had a normal diet and showed no significant abnormalities in behavior or appearance, and no deaths or significant toxic reactions were observed. After continuous observation for 14 days, the weight of mice in the control group and SBE group steadily increased without showing any significant differences. At the same time, there was no significant change in the organs of the mice in either group, and there was no significant difference in organ index (*p* > 0.05) (Figure 2).

### 2.3. Effects of SBE on Body Weight, DAI, and Colon Length of UC Mice

**Figure 3:**
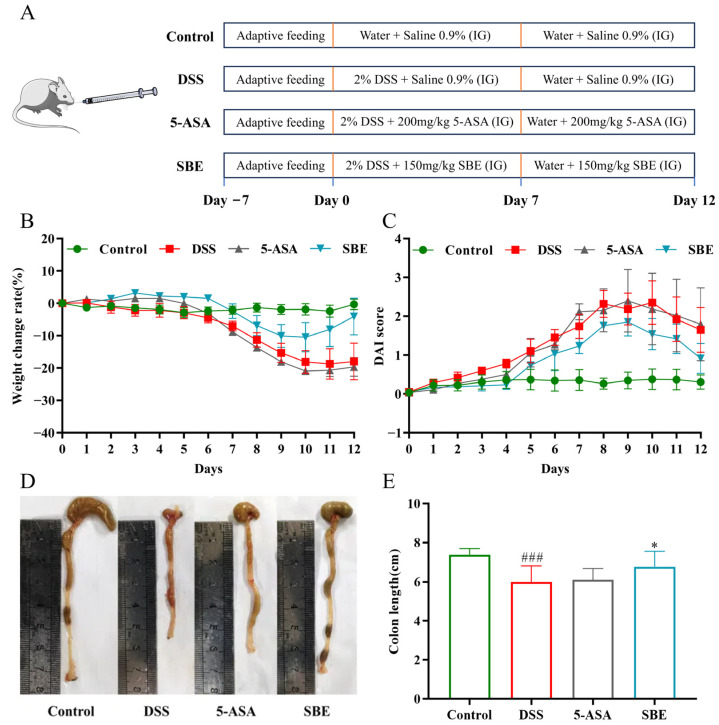
Effects of SBE on the pathological symptoms and the colon length of DSS-induced UC in mice. (**A**) Animal experiment design diagram; (**B**) body weight change of mice in each group; (**C**) DAI scoring index of mice in each group; (**D**) picture of the colon; (**E**) statistics of colon length in different treatment groups. 5-ASA, 200 mg/kg; SBE, 150 mg/kg. ###, *p* < 0.001; *, *p* < 0.05 (*n* = 6~8).

According to the experimental results, the weight change in mice in each group was relatively smooth for 6 days before the establishment of the injury model. From the 6th day of administration, the weight of mice in other groups dropped sharply, except in the control group. After 11 days of administration, the weight recovery of mice in each administration group was faster than that in the DSS group (Figure 3B). After DSS intervention, mice showed obvious symptoms such as diarrhea and hematochezia, and DAI in the SBE treatment group decreased compared with that in the DSS group (Figure 3C). At the same time, the effect of the SBE group was better than the 5-aminosalicylic acid (5-ASA) group, and the symptoms were relieved. The colon length of the DSS group was significantly shortened compared to the control group (*p* < 0.001), and all groups had a certain improvement effect on it. The SBE group had a significant effect (*p* < 0.05), and the improvement effect was better than that in the 5-ASA group (Figure 3D,E).

### 2.4. Effects of SBE on Colon Histopathological Changes in UC Mice

**Figure 4:**
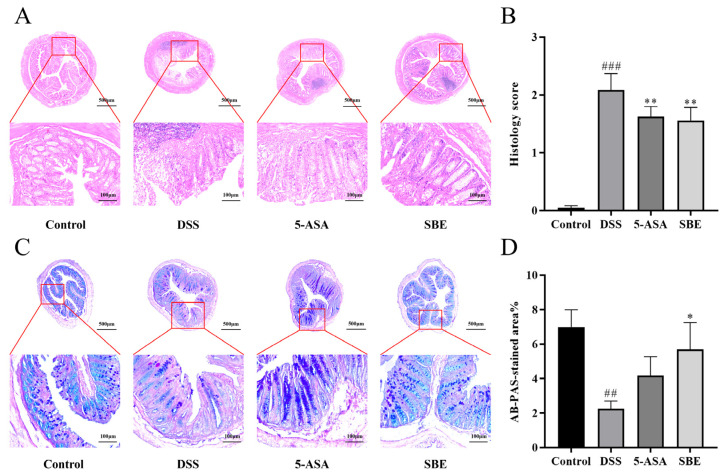
Effects of SBE on H&E-stained and AB-PAS-stained colon tissue sections of DSS-induced UC in mice. (**A**) HE-stained of representative tissue sections (40× magnification; 200× magnification); (**B**) histopathological scores; (**C**) AB-PAS-stained of representative tissue sections (40× magnification; 200× magnification); (**D**) grayscale analysis of AB-PAS-stained tissue sections. 5-ASA, 200 mg/kg; SBE, 150 mg/kg. ##, *p* < 0.01; ###, *p* < 0.001; *, *p* < 0.05; **, *p* < 0.01. (*n* = 6~8).

The results of histopathological analysis on the transverse section of the colon showed that crypts were destroyed in the colon tissue in the DSS group, and goblet cells were severely absent accompanied by inflammatory cell infiltration (Figure 4A). Compared with that in the DSS group, the degree of colon injury was significantly decreased in each administration group (*p* < 0.05) (Figure 4B). Goblet cells of colon tissue of mice in the control group were abundant and contained a large amount of mucopolysaccharides. The content of goblet cells was significantly reduced after DSS intervention. The absence of goblet cells and the reduction in mucopolysaccharide content were significantly reversed in the administration group of SBE (*p* < 0.05) (Figure 4C,D).

### 2.5. Effects of SBE on Inflammatory Factors and Oxidative Stress Indexes of UC Mice

**Figure 5:**
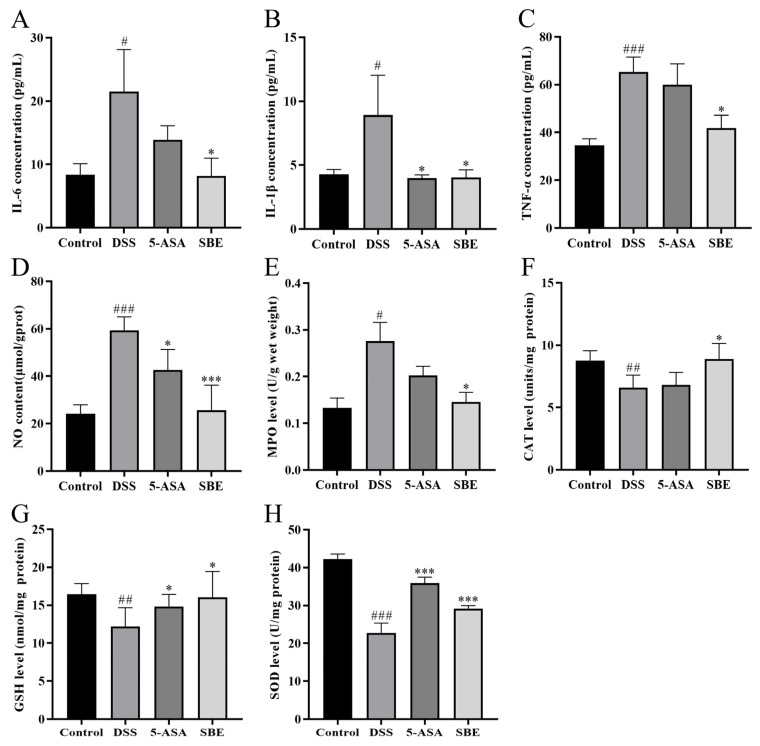
Effects of SBE on inflammatory factors and oxidative stress indicators levels of DSS-induced UC in mice. Blood serum IL-6 (**A**), IL-1β (**B**), and TNF-α (**C**) contents; colon tissue NO (**D**), MPO (**E**), CAT (**F**), GSH (**G**), and SOD (**H**) levels; 5-ASA, 200 mg/kg; SBE, 150 mg/kg. #, *p* < 0.05; ##, *p* < 0.01; ###, *p* < 0.001; *, *p* < 0.05; ***, *p* < 0.001. (*n* = 6~8).

As shown in Figure 5, the serum levels of IL-6, IL-1β, and TNF-α in mice after DSS intervention were significantly increased (*p* < 0.05), and the inflammatory cytokine levels of the SBE group decreased significantly (*p* < 0.05). The results showed that the MPO activity and NO concentration in colon tissue of mice in the DSS group were significantly increased compared with that in the control group (*p* < 0.05). The MPO and NO levels in the SBE group were significantly decreased (*p* < 0.001). The levels of total glutathione (GSH), catalase (CAT), and total superoxide dismutase (SOD) in the colon tissue of mice in the DSS group were significantly decreased (*p* < 0.01), while those in the SBE group were significantly increased (*p* < 0.05).

### 2.6. Effect of SBE on the mRNA Expression of Related Genes in the Colon Tissue of UC Mice

**Figure 6:**
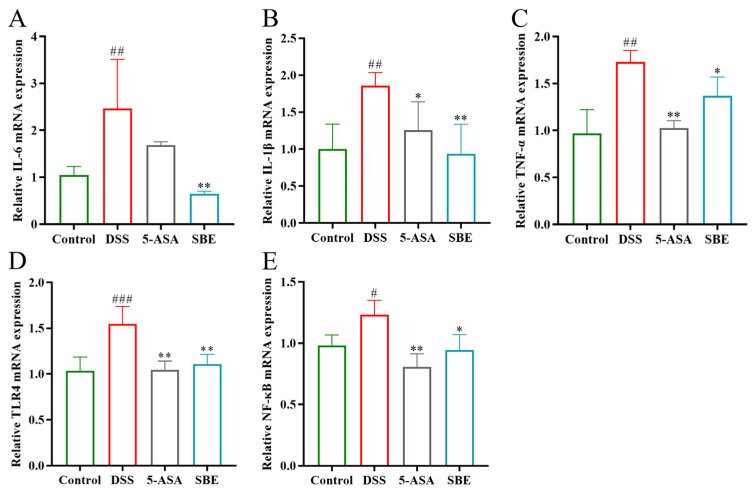
Effects of SBE on relative mRNA expression of inflammatory markers in DSS-induced UC in mice. (**A**) Relative IL-6 mRNA expression; (**B**) relative IL-1β mRNA expression; (**C**) relative TNF-α mRNA expression; (**D**) relative TLR4 mRNA expression; (**E**) relative NF-κB mRNA expression. 5-ASA, 200 mg/kg; SBE, 150 mg/kg. #, *p* < 0.05; ##, *p* < 0.01; ###, *p* < 0.001; *, *p* < 0.05; **, *p* < 0.01. (*n* = 3~4).

As shown in Figure 6, the relative mRNA expression levels of IL-6, IL-1β, TNF-α, TLR4, and NF-κB in the colon tissue of mice in the DSS group were significantly increased compared with those in the control group (*p* < 0.05), while the expression levels of related mRNA were significantly decreased in the SBE group (*p* < 0.05). These results suggest that SBE can reduce colon injury in UC mice by decreasing the expression of relevant mRNA.

### 2.7. Effects of SBE on Gut Microbiota Diversity of UC Mice

**Figure 7:**
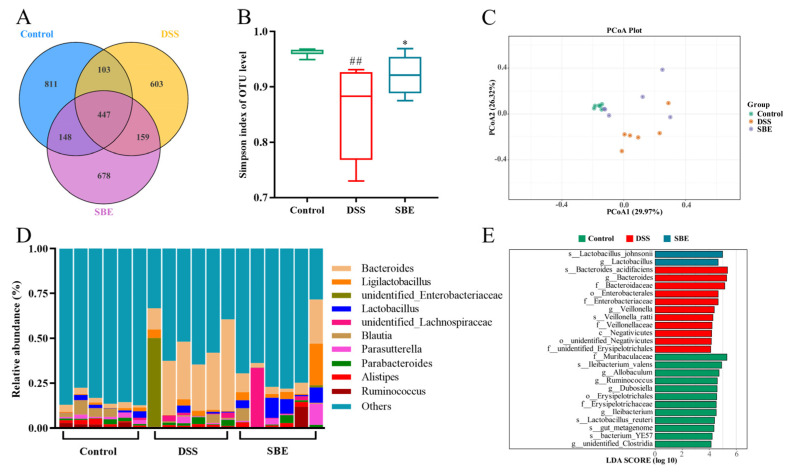
Effects of SBE on gut microbiota of DSS-induced UC in mice. (**A**) Venn diagram; (**B**) Simpson index; (**C**) ASV-based weighted UniFrac distance PCoA analysis; (**D**) genus-level bacterial communities form histograms; (**E**) ASV-based histogram of LDA value distribution. 5-ASA, 200 mg/kg; SBE, 150 mg/kg. ##, *p* < 0.01; *, *p* < 0.05. (*n* = 6).

A Venn diagram showed that 811, 603, and 678 microbial ASVs were detected in the control group, the DSS group, and the SBE group, respectively, and 447 ASVs were detected in the three groups (Figure 7A). Alpha diversity analysis showed that gut microbiota diversity and abundance were significantly reduced in the DSS group, which indicates that gut abundance was destroyed. Meanwhile, the Simpson index was significantly increased in the SBE treatment group (Figure 7B). The Beta diversity analysis of the gut microbes produced a PCoA map, which showed that the microbial community composition of the control and DSS groups was significantly different, while the SBE group was more similar to the control group (Figure 7C). Alpha and Beta diversity analysis showed significant differences in microbial composition between the control combination DSS groups. The abundance of microbial community at the genus level is shown in Figure 7D. Compared with those in the control group, the number of *Bacteroides* in the DSS group increased significantly, and the abundance of *parabacteroides* showed an upward trend. The abundances of *Ruminococcus* and *Parasutterella* decreased significantly, and the abundances of *Alistipes* showed a downward trend. Compared with the DSS group, SBE significantly reduced the abundance of *Bacteroides*. *Parabacteroides* abundance showed a decreasing trend while *Parasutterella*, *Alistipes*, and *Lactobacillus* in the SBE group had an increasing trend (Figure 7D). These findings suggest that DSS disrupts the homeostasis of gut microbiota, while SBE intervention restores the disturbance of gut microbiota to a certain extent.

LEfSe analysis was performed with the criterion of LDA > 4 to compare the microbial composition of each group for further determining the specific dominant microbiota associated with the SBE group. The results of the LDA histogram showed that *Bacteroides*, *Veillonella*, and *Veillonellaceae* were the main enrichment groups in the DSS group. The SBE group was mainly rich in *Lactobacillus* (Figure 7E). The dominant microbiota and the differential classification between the groups were shown, which reveals different enrichment classifications between the three groups and the presence of dysbiosis in the DSS group. Therefore, the increase in *Bacteroides* and *Veillonella* was the cause of UC in the DSS group, and the rise in lactic acid bacteria in the SBE treatment group could reduce gut inflammation.

**Figure 8:**
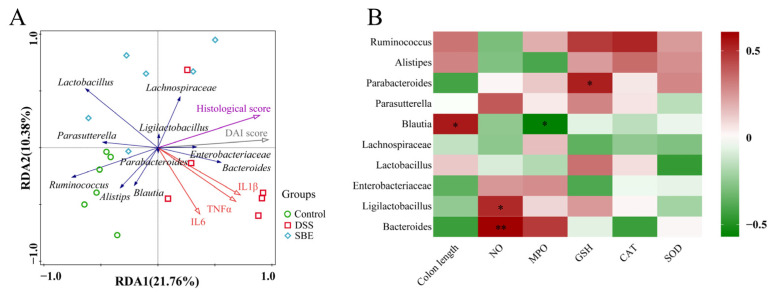
Effects of SBE on gut microbiota of DSS-induced UC in mice. (**A**) RDA analysis of inflammatory response markers at the genus level in each group; (**B**) correlation analysis of 10 dominant microflorae at genus level with environmental factors. 5-ASA, 200 mg/kg; SBE, 150 mg/kg. *, *p* < 0.05; **, *p* < 0.01. (*n* = 6).

The relationship between inflammatory factor markers and the gut microbiota was analyzed by redundancy analysis (RDA). The RDA results are shown in Figure 8A. *Bacteroides* in the DSS group were positively correlated with IL-6, IL-1β, and TNF-α, while *Lactobacillus* in the SBE group was negatively correlated with its three inflammatory markers. Among them, pathological tissue score and DAI index were positively correlated with the three inflammatory factor markers, positively correlated with *Bacteroides*, and negatively correlated with *Lactobacillus*. The relationship among the 10 bacteria with the highest abundance and colon length and oxidative stress factor was studied by correlation analysis. *Blautia* was significant positively correlated with colon length but significant negatively correlated with MPO level. *Bacteroides* and *Ligilactobacillus* were significant positively correlated with NO. *Parabacteroides* were significant positively correlated with GSH (Figure 8B).

## 3. Discussion

In recent years, natural products have played a great role in disease prevention and treatment, especially new types of natural products of plant [38]. These natural products have extremely high safety, among which the prebiotic effect of polyphenols can inhibit metabolic disorders and gut dysbiosis, and such compounds have become a potential therapeutic dietary strategy [39,40]. In addition, dietary fiber intake from medicinal and edible homologous plants may improve the production of short-chain fatty acids by altering the host microbiome, which enhances rodent-induced enteritis [41,42]. Zhou et al. investigated the therapeutic effect of maqui berry on UC, which can provide a research basis for the anti-UC of *S. bumalda* [43]. *S. bumalda*, as a rare edible shrub in China, has the characteristics of low toxicity and low cost. It is rich in various nutrients, and its tender leaves and flower buds contain flavonoids. According to the relevant literature, Staphyleaceae has anti-inflammatory, antibacterial, antioxidant, and anti-cancer biological activities [44,45]. Thus, the potential effects of *S. bumalda* on UC mice were investigated in this study.

In this study, SBE has shown good safety in mouse experiments, and will not adversely affect animals at the maximum tolerated dose (20 g/kg). At this dose, the body weight of each group of mice increased steadily, and there was no significant difference in organ color and organ index between the treatment group and the control group. This good security lays a solid foundation for its subsequent functional development. SBE alleviated the symptoms of weight loss, diarrhea symptoms, and fecal occult blood of UC mice. It reduced the histopathological section score. During this process, we found that the cecum of mice in the DSS group was atrophic compared with that in other groups. We speculated that this phenomenon was related to the severe impact of colon injury on the food intake of mice. At the same time, the level of inflammatory cytokines and oxidative stress showed an improvement effect on UC mice. However, which active ingredient played a role in *S. bumalda* is unclear. In the previous component analysis, we identified 19 compounds, including four major flavonoids, hyperoside, rutin, isorhoifolin, and kaempferol-3-O-rutinoside in SBE, all of which have certain anti-inflammatory effects. Among them, hyperoside is the main component in SBE. According to previous research, hyperoside can improve DSS-induced colitis through MKRN1-mediated PPARγ signaling and Th17/Treg balance regulation [46]. In addition, rutin can alleviate DSS-induced ulcerative colitis in mice through p38/MK2 and PI3K/Akt/GSK3β/NF-κB pathways, maintaining intestinal integrity and balancing the cytokine ratio [47]. Kaempferol-3-O-rutinoside can down-regulate the expression of TNF-α and IL-6 in LPS-induced RAW264.7 cells [48]. In this study, SBE rich in hyperoside can significantly reduce DAI changes, colon shortening, and histological changes induced by DSS; in addition, it can inhibit inflammatory response and reduce the expression levels of TNF-α and IL-6. Hyperoside also has some antioxidant effects [46], and these results are consistent with our experimental results. Therefore, we speculate that hyperoside is the main component of SBE that plays an anti-inflammatory role in this study. Flavonoids dominated by hyperoside may be the pharmacological basis for SBE to exert its anti-inflammatory activity.

The main manifestations of UC induced by DSS are weight loss, bloody diarrhea, ulcer formation, decreased epithelial cells in colon tissue, and infiltration of leukocyte layer [49]. The main changes in the upper cell layer include the proliferation of crypt epithelial cells and the absence of goblet cells. Connective tissue repair is the hallmark of granulosa tissue, consisting mainly of CD31^+^ endothelial cells, myofibroblasts, iNOS^+^ macrophages, and neutrophils, as well as new capillaries composed of lymphocytes and cell fragments [50]. The experimental results of this study showed that in the DSS model group, there was extensive aggregation of inflammatory cells in the colon submucosa, accompanied by significant inflammatory cell infiltration, serious recess destruction, and serious loss of goblet cells. In addition, the mRNA expression levels of IL-6, IL-1β, TNF-α, and NF-κB were significantly increased. The related inflammatory reactions were reversed to a certain extent after SBE intervention. The results of a previous experiment showed that artemisinin analogue SM934 can reduce the mRNA expression of pro-inflammatory cytokines in DSS-induced UC mice, as well as the percentages of macrophages and neutrophils in colon tissue. In addition, the production of pro-inflammatory mediators in RAW264.7 cells and THP-1-derived macrophages and the activation of the NF-κB signaling pathway stimulated by LPS in vitro can be inhibited after drug intervention [51]. And our experimental results are highly similar, so we speculate that the protective effect of SBE on UC mice may be attributed to its inhibitory effect on neutrophils and macrophages, as well as its inhibitory effect on NF-κB signaling. TLR is a key factor of the innate immune system [52], and NF-κB is an important inflammatory regulator given that it mediates the transcription and expression of TNF-α, IL-1β, IL-6, and other inflammatory mediators and growth factors [53]. In this study, relevant mRNA expression levels in the colon tissues of mice were measured. In the DSS group, NF-κB and TLR4 were overexpressed, and IL-6, IL-1β, and TNF-α were activated at the same time. Meanwhile, relevant mRNA expression levels were decreased in the SBE treatment group. As previously reported, Zanthoxylum peel extract can inhibit the expression of TNF-α, IL-1β, and IL-12 by regulating TLR4 and TLR4-related pathways in experimental colitis induced by DSS in mice and LPS-induced inflammation in J774.1 cells [54]. The results are quite similar to the findings of this study. Therefore, SBE inhibits TLR4/NF-κB signaling pathway, which alleviates DSS-induced UC. This deduction needs to be further verified by Western blot assay.

The gut microbiota is essential in the human microbiome and is a rich and stable ecosystem. The disturbance of the gut microbiota is implicated in the pathogenesis of UC [55]. In this study, the relative abundance of *Bacteroides* in the DSS treatment group increased significantly, which was consistent with the previous results of Seth M. Bloom et al. [56]. Moreover, the abundance of *Ruminococcus* and *Parasutterella* decreased significantly. Hu Zhao et al. studied arecoline aggravating acute UC in mice by affecting gut microbiota and serum metabolites. The results showed that, compared with that in the control group, the abundance of *Parasutterella* in the DSS group decreased, that *Parasutterella* significantly decreased in the treatment group compared with that in the DSS group, and that *Parasutterella* played a similar role in the homeostasis of gut microbiota as described in this study [57]. The relative abundance of *Bacteroides* in the SBE administration group decreased significantly, while the relative abundance of *Lactobacillus* showed a certain upward trend. *Lactobacillus* is a recognized bacterium that can produce SCFAs, and a decreased proportion of *Lactobacillus* is closely related to the occurrence of UC [58]. As previously reported, salidroside alleviates dextran sulfate sodium-induced colitis in mice by modulating the gut microbiota, in which the treatment group significantly increases the abundance of *Lactobacillus*. The findings of that study are consistent with the experimental results [59]. Therefore, SBE alleviates UC by gradually restoring homeostasis of gut microbiota through increasing *Lactobacillus*. The results of LEfSe analysis showed that *Bacteroides* and *Veillonella* were enriched in the DSS treatment group, and *Lactobacillus* was mainly enriched in the SBE treatment group, which was in good agreement with the statistics of the relative abundance of microflora. In addition, we analyzed the correlation between the 10 dominant microbiota and environmental factors at the genus level. The RDA results showed that *Bacteroides* was positively correlated with IL-6, IL-1β, and TNF-α, and *Lactobacillus* was negatively correlated with it. From these results, we speculated that the increase in the relative abundance of *Bacteroides* may lead to the occurrence of UC. Furthermore, the SBE administration group could improve the colon inflammation in mice by increasing the relative abundance of *Lactobacillus*.

## 4. Materials and Methods

### 4.1. Materials and Reagents

The samples used in the experiment were collected from Datong Town, Qichun County, Huanggang City, Hubei Province, China, and the samples were identified by Dr. Gao Zhou from Hubei University of Technology to be the tender leaves and buds of *S. bumalda*. The certificate specimen (serial number, 20210701) and extract were preserved in the refrigerator in the laboratory. The extraction process of the samples involved crushing and sieving the dried tender leaves and flower buds of *S. bumalda*, mixing them with 70% ethanol at a ratio of 1:10 for ultrasonic extraction. This process was repeated three times, with each extraction lasting for 30 min. The filtrate from the three extractions was combined, filtered, and concentrated into a paste like extract using a rotary evaporator to obtain SBE. The extraction rate was 22.41%. Mouse monocyte macrophage RAW264.7 (No. GDC0143) was purchased from the China Center for Type Culture Collection (Wuhan University, Wuhan, China); DSS(9011-18-1) was purchased from MP Biomedicals in the United States; interleukin 1β (IL-1β, SEKM-0002), interleukin-6 (IL-6, SEKM-0007), and tumor necrosis factor-α (TNF-α, SEKM-0034) quantitative enzyme-linked immunosorbent assay (ELISA) were purchased from Beijing Solebao Technology Co., Ltd., Beijing, China; the MPO test kit (A004-1-1) and NO assay kit (A012-1-2) were purchased from Nanjing Jiancheng Institute of Biotechnology; the SOD Assay Kit (S0101S), CAT Assay Kit (S0051), and GSH detection kit (S0052) were purchased from Shanghai Biyuntian Biotechnology Co., Ltd., Shanghai, China. Chromatographic grade methanol and acetonitrile were purchased from Thermo Fisher Technologies. Other reagents used in this experiment were of analytical grade.

### 4.2. Experimental Animal

A total of 40 SPF grade ICR mice (5~6 weeks old, weight 18~22 g) were selected for acute toxicity test, half male and half female, which were purchased from Henan Skobes Biotechnology Co., Ltd (SCXK (Y) 2020-0005, Anyang, China). A total of 32 SPF grade male C57BL/6J mice (6–8 weeks old, weight: 20~25 g) were selected for the study of anti-UC activity, and purchased from Hubei Experimental Animal Research Center (SCXK (E) 2020-0018, Wuhan, China). The temperature and humidity of the mouse breeding environment were (22 ± 2) °C and (55 ± 5) %, with 12 h of light and 12 h of dark circulation. The experiment began after 7 days of adaptive feeding. This research subject conformed to the “Regulations on the Management of Experimental Animals of Hubei Province” and the “Articles of Association of Research Ethics and Technical Safety Committee of the Hubei University of Technology”, with the ethics batch number of HBUT20230076.

### 4.3. Experimental Method

#### 4.3.1. Qualitative and Quantitative Analysis of the Main Components in SBE by HPLC and LC/MS Methods

The SBE was dissolved in 0.1% formic acid water and methanol in a ratio of 1:1 by ultrasound, and the subsequent filtrate was filtered by 0.22 µm microporous filter membrane to obtain the test sample; the sample was first separated and analyzed by HPLC, then analyzed by LC-MS for its chemical components. According to the analysis results, the components were qualitatively and quantitatively analyzed by HPLC under specific conditions. The HPLC conditions are: 5C18-AR-Ⅱ column, mobile phase A: 0.1% formic acid aqueous solution; mobile phase B: acetonitrile solution, gradient elution: 0–5 min, 10% B; 5–20 min, 10–20% B; 20–25 min, 20–22% B; 25–30 min, 22–25% B; 30–35 min, 25–40% B; 35–40 min, 40–65% B; 40–45 min, 65% B; 45–47 min, 65–10% B; 47–50 min, 10% B. Flow rate of 1mL/min, column temperature of 25 °C, injection volume of 10 µL, and detection wavelength of 254 nm. The chromatographic column for LC-MS analysis was an Eclipse Plus C18 (RRHD) 1.8 µm column; 0.1% formic acid water (A) and 0.1% formic acid methanol (B) were used as eluents. The proportion of organic phase increased from 10% to 90% within 12 min and gradually recovered to 10% within 12 min to 15 min. The sample volume was 5 µL and the flow rate was 0.2 mL/min. The content of each chemical component was calculated by the standard curve of the reference substances.

#### 4.3.2. Acute Toxicity Study of SBE in ICR Mice

At the beginning of the experiment, we preliminarily evaluated the safety and anti-inflammatory activity of SBE through cytotoxicity tests (Figure S1), and on this basis, we conducted an acute toxicity test in mice. A total of 40 ICR mice were randomly divided into two groups: the control group and the SBE group, with 20 mice in each group, consisting of half males and half females. Mice were fasted for 12 h before the experiment. The SBE group was given a dose of 20 g/kg (the maximum dose), and both groups were given two times of gavage with the total volume of 0.4 mL/10g, with an interval of 5 h. The control group was given the same volume of normal saline. The status of the mice was observed continuously for 4 h after administration, then once a day for 14 days. The weight change, any deaths, and signs of poisoning reactions in the mice were recorded. After the 14-day observation period, the mice were sacrificed under carbon dioxide anesthesia, then subjected to a gross anatomical examination to observe whether there were significant changes in the organs, and the organ index of the mice was calculated simultaneously.

#### 4.3.3. Animal Experiments and Dosing Determination

As shown in Figure 3A, 32 C57BL/6J mice were randomly divided into four groups, namely, the control group, the DSS group, the positive control group of 5-ASA (200 mg/kg), and the SBE group (150 mg/kg), with 8 mice in each group. The human dosage of the *S. bumalda* herb recorded in Chinese Materia Medica is 9–15 g, and the dosage for mouse administration is calculated to be 266.95–444.20 mg/kg according to the dosage of the ethanol extract. Based on the preliminary experimental results, the final dosage for administration is determined to be 150 mg/kg. The control group fed and drank normally. All other groups of mice freely drank the prepared 2% DSS aqueous solution for 7 days to establish a UC model. The drug treatment time was 13 days. On the 8th day, each group of mice was given free drinking distilled water. The treatment group was given 0.1 mL/10 g of the drug dose by gavage, while the control group was given an equal volume of normal saline. The mice were supplemented daily with purified water and a newly formulated DSS solution. All mice were euthanized on the second day after the last administration.

#### 4.3.4. Evaluation of DAI

During the experiment, mice were weighed every day before drug treatment, and the ratio of weight before each drug administration to the initial weight was calculated. The average weight change rate (%) of mice was obtained, and the occult blood in the stool of mice was detected by O-toluidine method. Fecal viscosity of mice was observed and recorded every day for DAI score [60]. The DAI score is calculated as follows: DAI score = (weight change score + fecal viscosity score + fecal occult blood score)/3. The DAI scoring criteria are shown in Table S2.

#### 4.3.5. Colonic Histological Analysis

The gut contents were washed clean with precooled saline, and the condition of colonic mucosal lesions was observed. Approximately 1 cm of colonic tissue in the same area of each mouse was cut and fixed with 4% paraformaldehyde solution, which was used for paraffin embedding and section, and colon tissue sections were stained with hematoxylin and eosin (H&E) and Alcian Blue/Periodic acid–Schiff (AB-PAS), observed under a microscope, and the degree of tissue injury was scored. H&E staining [43] was used to evaluate pathological damage to the colon. One of the characteristics of UC was a protective layer mucus defect, which was attributed to a reduction in the number of goblet cells; AB-PAS staining [61] was used to observe the number of goblet cells and mucous content in the colon. The histological scoring criteria related to colitis are shown in Table S3.

#### 4.3.6. Determination of Inflammatory Factors in Blood Serum

The serum frozen in advance was removed from the −80 °C refrigerator and divided into different packages. The contents of IL-6, IL-1β, and TNF-α in the serum of mice in each group were determined according to the instructions of the ELISA kit.

#### 4.3.7. Determination of MPO, NO, GSH, CAT, and SOD Levels in Colon Tissue

An appropriate amount of colon tissue was weighed and homogenized with normal saline. The protein concentration of homogenate was determined by the Coomassie Brilliant Blue method, then the levels of MPO, NO, GSH, CAT, and SOD in the colon tissue were detected according to the kit instructions.

#### 4.3.8. Detection of mRNA Expression of Related Genes in Colon Tissue-Related Signaling Pathways by RT-qPCR

The samples were prepared by mixing appropriate amount of mouse colon tissue according to groups. Trizol was used to prepare colon tissue homogenate on ice using a tissue homogenizer, total RNA was extracted, and RNA reverse transcription synthesis was performed using a HiFiScript cDNA Synthesis Kit. UltraSYBR mixture was prepared for quantitative analysis of the real-time PCR reaction system. The cycle threshold (Ct) of each gene was recorded and normalized with the Ct value of β-actin by the 2^−∆∆Ct^ method. The primer design of RT-qPCR was shown in Table S4.

#### 4.3.9. Analysis of Gut Microbiota

After the mice were euthanized, the colon contents were rinsed with sterile water, and the colon contents of the control group, DSS group, and SBE group were collected for 16S rRNA sequencing. Total genome DNA from samples was extracted using the CTAB method. DNA concentration and purity were monitored on 1% agarose gel. According to the concentration, DNA was diluted to 1 ng/L using sterile water. 16S rRNA/18S rRNA/ITS genes of distinct regions (16S V4/16S V3/16S V3-V4/16S V4-V5, 18S V4/18S V9, ITS1/ITS2, Arc V4) were amplified used specific primer with the barcode. A sequencing library was generated using the truseq^®^DNA PCR-Free Sample Preparation Kit (Illumina, San Diego, CA, USA) and an index code was added. Finally, the library was sequenced on the Illumina NovaSeq platform. Sequence analysis was performed by Uparse software (Uparse v7.0.1001), and sequences with 97% similarity were assigned to the same OTUs. The representative sequence for each OTU was screened for further annotation. The Simpson index was calculated using QIIME for its Alpha diversity analysis and is displayed in R software (Version 4.1.2). Principal coordinate analysis (PCoA) was used to obtain principal coordinates from multidimensional data and visualize them for Beta diversity analysis.

#### 4.3.10. Statistical Analysis

Data are expressed as the mean ± SEM. Statistical analysis was conducted using IBM SPSS Statistics 26 software. The body weight, DAI score, colon length, biochemical indicators in colon tissue, and inflammatory factor levels in colon tissue of each group of mice were found to follow a normal distribution. Single-factor variance analysis was used to compare the sample mean between multiple groups, and a *t*-test was used to compare the sample mean pairwise between groups. The difference is statistically significant with *p* < 0.05.

## 5. Conclusions

In this study, we aimed to investigate the anti-inflammatory activity of SBE in inflammatory bowel disease and evaluate its safety. The experimental results showed that SBE exhibited good safety in mouse acute toxicity experiments, reducing the expression of pro-inflammatory factors, demonstrating anti-inflammatory effects at multiple levels, and exerting a regulatory effect on the imbalance of gut microbiota. Therefore, *S. bumalda* can be used as a low toxic natural product for adjunctive treatment of inflammatory bowel disease.

## Figures and Tables

**Table 1:** LC-MS analysis of components in SBE.

No.	Rt (min)	*m*/*z*	Identification	Formula	Ion Mode
1	5.39	152.03456	3,4-Dihydroxybenzoic acid [21]	C_7_H_6_O_4_	−
2	5.55	239.05609	Eucomic acid [22]	C_11_H_12_O_6_	−
3	5.58	399.09357	Sinapinic acid-O-glucuronide [23]	C_17_H_20_O_11_	−
4	6.34	465.10275	Hyperoside [24]	C_21_H_20_O_12_	+
5	6.35	609.14667	Rutin [25]	C_27_H_30_O_16_	−
6	6.51	447.09328	Quercitrin [26]	C_21_H_20_O_11_	−
7	6.65	191.03455	Scopoletin [27]	C_10_H_8_O_4_	−
8	7.00	330.13217	Tricin [28]	C_17_H_14_O_7_	+
9	7.45	318.29907	Myricetin [29]	C_15_H_10_O_8_	+
10	7.75	478.10577	Isorhamnetin-3-O-β-D-Glucoside [30]	C_22_H_22_O_12_	−
11	7.77	434.15781	Apigenin 7-O-β-glucoside	C_21_H_20_O_10_	+
12	8.53	293.21255	Gingerol [31]	C_17_H_26_O_4_	−
13	8.90	400.30431	Nobiletin [32]	C_21_H_22_O_8_	+
14	8.94	488.35620	Asiatic acid [33]	C_30_H_48_O_5_	+
15	8.96	576.40784	Isorhoifolin [34]	C_27_H_30_O_14_	+
16	9.25	559.31256	Apiin [35]	C_26_H_28_O_14_	−
17	9.99	503.32520	Medicagenic acid [36]	C_30_H_46_O_6_	+
18	10.07	379.23434	Gingerdiol-3,5-diacetate [37]	C_21_H_32_O_6_	−
19	10.28	592.38171	Kaempferol-3-O-rutinoside [25]	C_27_H_30_O_15_	+

## Data Availability

Data are contained within the article and Supplementary Materials.

## Supplementary Materials

The following supporting information can be downloaded at: https://www.mdpi.com/article/10.3390/molecules29215030/s1, Table S1: Methodology verification of four main components in SBE; Table S2: DAI scoring criteria; Table S3: Histological scoring criteria related to colon inflammation; Table S4: Primer sequences for quantitative real-time PCR reactions; Figure S1. Cytotoxicity experiments of SBE and anti-inflammatory effects in vitro. (A) Effects of different concentrations of SBE on RAW 264.7 cells viability; (B) inhibition of NO production induced by LPS. ###, *p* < 0.001; **, *p* < 0.01; ***, *p* < 0.001.
