# Supplementary material for: Staphylea bumalda Alleviates Dextran Sulfate Sodium-Induced Ulcerative Colitis in Mice by Regulating Inflammatory Cytokines, Oxidative Stress, and Maintaining Gut Homeostasis
Source: Molecules. 2024 Oct 24;29(21):5030. doi: 10.3390/molecules29215030 (PMC11547842; doi:10.3390/molecules29215030)
Supplement: Supplementary file 1 [file molecules-29-05030-s001.zip › molecules-3248519-supplementary.pdf]

Staphylea bumalda alleviates dextran sulfate sodium-induced ulcerative colitis in mice by regulating inflammatory cytokines, oxidative stress, and maintaining gut homeostasis

Lu Wang [1], Sha Long [1], Qi Zeng [1], Wanrong Dong [1], Yaoyao Li [1], Jiangtao Su [1], Yuxin Chen [1,2], Gao Zhou [*1,2,3]

1 Hubei Key Laboratory of Industrial Microbiology, Key Laboratory of Fermentation Engineering (Ministry of Education), Cooperative Innovation Center of Industrial Fermentation (Ministry of Education & Hubei Province), Hubei University of Technology, Wuhan 430068, China
2 National "111" Center for Cellular Regulation and Molecular Pharmaceutics, School of Life and Health Sciences, Hubei University of Technology, Wuhan 430068, China
3 Post-doctoral Research Center of Mayinglong Pharmaceutical Group Co., Ltd., Wuhan, Hubei 430064, China
* Correspondence: drzhougao@foxmail.com

Supplement Table Captions

Table S1 Methodology verification of four main components in SBE

Compound	Calibration curve	R^2	RSD (%)		
			precision	stability	repeatability
rutin	y = 0.1646x + 0.006	0.9999	0.19	2.44	0.70
hyperoside	y = 0.207x - 0.0404	0.9998	0.04	0.75	0.20
kaempferol-3-O-rutinoside	y = 0.039x + 0.0061	0.9999	0.05	4.47	3.39
isorhoifolin	y = 0.0824x + 0.0236	0.9999	0.09	4.01	1.49

Table S2 DAI scoring criteria

score	Weight loss rate	fecal viscosity	Fecal occult blood
0	Normal	Normal	No change
1	1% ~ 5%	The feces are loose and soft	Light blue color in 1-3 minutes
2	5% ~ 10%	Fecal sticky tail	Gradually turning blue from 0-60s
3	10% ~ 15%	Hematochezia	Immediately turn blue
4	>15%	Bloodstained tail	Immediately turn deep blue

Table S3 Histological scoring criteria related to colon inflammation

Score	Severity of inflammation	Degree of crypt loss	Degree of ulcer
0	Normal	Normal	Normal
1	Slight inflammatory cell infiltration	1/3 crypt loss	1-2 ulcer lesions
2	Inflammatory cells extend into the submucosa	2/3 crypt loss	3-4 ulcer lesions
3	Inflammatory infiltration extends across the wall	The entire crypt is lost	Widespread ulcer

Table S4 Primer sequences for quantitative real-time PCR reactions

Gene	Forward (5‘-3’)	Reverse (5’-3’)
IL-1β	GCAACTGTTCCTGAACTCAACT	ATCTTTTGGGGTCCGTCAACT
IL-6	CCAAGAGGTGAGTGCTTCCC	CTGTTGTTCAGACTCTCTCCCT
TNF-α	CCCTCACACTCAGATCATCTTCT	GCTACGACGTGGGCTACAG
TLR4	GCTTGAATCCCTGCATAGAGGTAG	CTTCAAGGGGTTGAAGCTCAGAT
NF-κB	TGGGAAACCGTATGAGCCTG	ATCCCGGAGTTCATCTCATAGT
β-actin	GGCTGTATTCCCCTCCATCG	CCAGTTGGTAACAATGCCATGT

Supplement Figure Captions

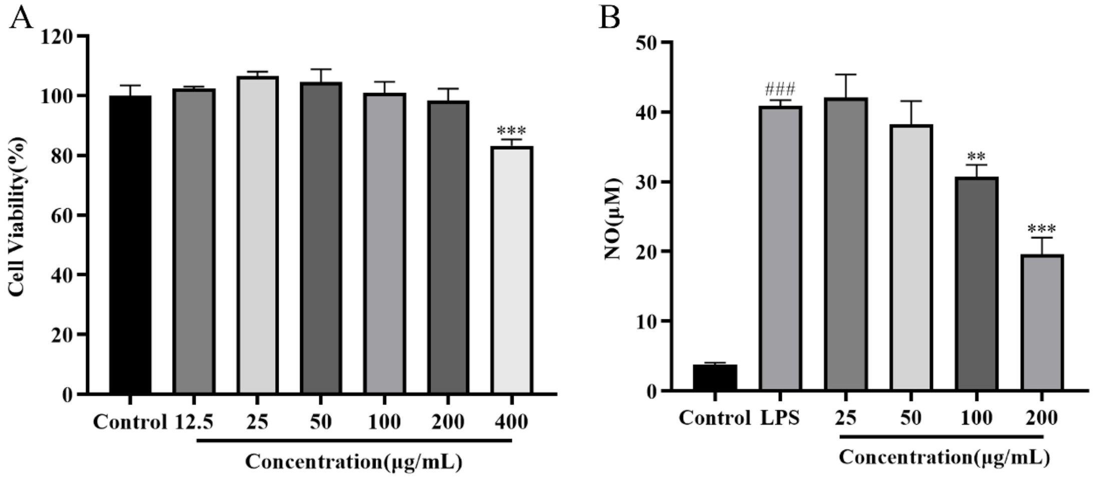

Figure S1. Cytotoxicity experiments of SBE and anti-inflammatory effects *in vitro*. (A) Effects of different concentrations of SBE on RAW 264.7 cells viability; (B) Inhibition of NO production induced by LPS. ###, $p < 0.001$; **, $p < 0.01$; ***, $p < 0.001$.
